# Local Gene Delivery System by Bubble Liposomes and Ultrasound Exposure into Joint Synovium

**DOI:** 10.1155/2011/203986

**Published:** 2011-04-28

**Authors:** Yoichi Negishi, Yuka Tsunoda, Yoko Endo-Takahashi, Yusuke Oda, Ryo Suzuki, Kazuo Maruyama, Matsuo Yamamoto, Yukihiko Aramaki

**Affiliations:** ^1^Department of Drug and Gene Delivery Systems, School of Pharmacy, Tokyo University of Pharmacy and Life Sciences, 1432-1 Horinouchi, Hachioji, Tokyo 192-0392, Japan; ^2^Department of Biopharmaceutics, Teikyo University, 1091-1 Suwarashi, Midori-ku, Sagamihara, Kanagawa 252-5195, Japan; ^3^Department of Periodontology, Showa University School of Dentistry, 2-1-1 Kitasenzoku, Ohta-ku, Tokyo 145-8515, Japan

## Abstract

Recently, we have developed novel polyethylene glycol modified liposomes (bubble liposomes; BL) entrapping an ultrasound (US) imaging gas, which can work as a gene delivery tool with US exposure. In this study, we investigated the usefulness of US-mediated gene transfer systems with BL into synoviocytes *in vitro* and joint synovium *in vivo*. Highly efficient gene transfer could be achieved in the cultured primary synoviocytes transfected with the combination of BL and US exposure, compared to treatment with plasmid DNA (pDNA) alone, pDNA plus BL, or pDNA plus US. When BL was injected into the knee joints of mice, and US exposure was applied transcutaneously to the injection site, highly efficient gene expression could be observed in the knee joint transfected with the combination of BL and US exposure, compared to treatment with pDNA alone, pDNA plus BL, or pDNA plus US. The localized and prolonged gene expression was also shown by an *in vivo* luciferase imaging system. Thus, this local gene delivery system into joint synovium using the combination of BL and US exposure may be an effective means for gene therapy in joint disorders.

## 1. Introduction

Intra-articular gene therapy is considered a feasible technique to deliver therapeutic proteins to suppress inflammation and destruction of the joints in rheumatoid arthritis and osteoarthritis, because it could minimize extra-articular adverse effects linked to the systemic injection of drugs [1, 2]. To achieve successful gene therapy in a clinical setting, it is critical that the gene delivery system is safe, easy to apply, and provides therapeutic transgene expression. Previous studies using viral vectors reported the successful transfer of therapeutic genes into the target cells in joint diseases [1, 2], but because of the considerable immunogenicity related to the use of viruses, nonviral gene transfer still needs to be developed [3]. Recently, it has been reported that therapeutic ultrasound as a physical non-viral gene transfer method enables genes to permeate cell membranes. Acoustic cavitation is involved in the mechanism of gene transfer [4–8]; however, to achieve efficient gene transfer, high intensity ultrasound (US) is needed, leading to tissue damage [9–11]. In contrast, low-intensity US in combination with microbubbles has recently acquired much attention as a safe method of gene delivery [12–16]; however, microbubbles have problems with size, stability, and targeting function. Liposomes have been known as drug, antigen, and gene delivery carriers [17–21]. To solve the above-mentioned issues of microbubbles, we previously developed polyethylene glycol- (PEG-) modified liposomes entrapping echo contrast, bubble liposomes (BL), which can function as a novel gene delivery tool by applying them with US exposure [22–27]. 

 The establishment of a method to deliver genes into joints by the combination of BL and US exposure may facilitate the development of a safe and efficient gene therapy for joint disorders. In the present study, we investigated the usefulness of US-mediated gene transfer systems with BL into synoviocytes *in vitro* and the joint synovium *in vivo*.

## 2. Materials and Methods

### 2.1. Preparation of Bubble Liposomes

Bubble liposomes were prepared by the previously described methods [22, 23, 26]. Briefly, PEG liposomes composed of 1,2-dipalmitoyl-*sn*-glycero-3-phosphocholine (DPPC) (NOF Corporation, Tokyo, Japan) and 1,2-distearoyl-*sn*-glycero-3-phosphatidyl-ethanolamine-polyethylen eglycol (DSPE-PEG_2000_-OMe) (NOF Corporation) in a molar ratio of 94 : 6 were prepared by a reverse phase evaporation method. In brief, the reagents were dissolved 1 : 1 (v/v) in chloroform/diisopropyl ether. Phosphate-buffered saline was added to the lipid solution, and the mixture was sonicated and then evaporated at 47°C. The organic solvent was completely removed, and the size of the liposomes was adjusted to less than 200 nm using extruding equipment and a sizing filter (pore size: 200 nm) (Nuclepore Track-Etch Membrane, Whatman plc, UK). The lipid concentration was measured using a Phospholipid C test Wako (Wako Pure Chemical Industries, Ltd., Osaka, Japan). BL were prepared from liposomes and perfluoropropane gas (Takachio Chemical Ind. Co. Ltd., Tokyo, Japan). First, 2 mL sterilized vials containing 0.8 mL liposome suspension (lipid concentration: 1 mg/mL) were filled with perfluoropropane gas, capped, and then pressurized with a further 3 mL perfluoropropane gas. The vial was placed in a bath-type sonicator (42 kHz, 100 W) (BRANSONIC 2510j-DTH; Branson Ultrasonics Co., Danbury, Conn, USA) for 5 min to form BL.

### 2.2. Plasmid DNA

The plasmid pCMV-Luc is an expression vector encoding the firefly luciferase gene under the control of a cytomegalovirus promoter. The plasmid pDsRed-Express-N1 (Clontech Laboratories, Inc., Mountain View, Calif, USA) is an expression vector encoding the red fluorescent protein under the control of a cytomegalovirus promoter.

### 2.3. Transfection of Plasmid DNA into Primary Synoviocytes Using Bubble Liposomes

Primary synoviocytes (HFLS), which are primary fibroblast-like cells derived from the inflamed synovial tissue of rheumatoid arthritis patients, were purchased from Cell Applications, Inc. (San Diego, Calif, USA). The culture was performed according to the manufacturer's instructions. The day before transfection, cells (3 × 10^4^) were seeded in the wells of a 48-well plate (ASAHI TECHNOGLASS CO., Chiba, Japan). Five micrograms of pDNA and 60 *μ*g BL were mixed together with culture medium containing 10% FBS and added to the cells. The cells were immediately exposed to US (frequency, 2 MHz, duty, 50%; burst rate, 2.0 Hz; intensity, 2.5 W/cm^2^) for 10 sec through a 6-mm diameter probe placed in the well. A Sonopore 3000 (NEPA GENE, Co., Ltd., Chiba, Japan) was used to generate the US. The cells were washed twice with culture medium and cultured for two days. The cell lysate was prepared with lysis buffer (0.1 M Tris-HCl (pH 7.8), 0.1% Triton X-100, and 2 mM EDTA). Luciferase activity was measured using a luciferase assay system (Promega, Madison, WI) and a luminometer (LB96V; Belthold Japan Co. Ltd., Tokyo, Japan). The activity is indicated as relative light units (RLU) per mg of protein.

### 2.4. Transfection of Plasmid DNA with Lipofectamine 2000

The day before transfection, HFLS-RA (4 × 10^4^) were seeded in the wells of a 48-well plate (ASAHI TECHNOGLASS CO., Chiba, Japan). Then, 0.25 *μ*g pDNA (final concentration, 25 nM) wsa diluted in Opti-MEM (GIBCO). Next, 1.25 *μ*g Lipofectamine 2000 (LF2000) (Invitrogen Japan K.K., Tokyo, Japan) was diluted in Opti-MEM. These solutions were mixed and added to the cells. After 4 and 24 hours, the cells were washed with PBS and cultured for two days. The experiments were performed according to the manufacturers' instructions.

### 2.5. Measurement of Luciferase and DsRed Expression

Cell lysate was prepared with lysis buffer (0.1 M Tris-HCl (pH 7.8), 0.1% Triton X-100, and 2 mM EDTA). Luciferase activity was measured using a luciferase assay system (Promega, Madison, WI) and a luminometer (LB96V; Belthold Japan Co. Ltd.). The activity is indicated as relative light units (RLU) per mg protein. To analyze DsRed expression, the treated cells were observed with a fluorescence microscope (Axiovert 200 M; Carl Zeiss).

### 2.6. In Vivo Gene Delivery into the Joint Synovium of Mice with Bubble Liposomes and Ultrasound Exposure

 To determine the efficiency of gene delivery, animals were divided into five experimental groups and one control group (*n* = 4 in each group). ICR mice (5 weeks old, male) were anesthetized with an *i.p.* injection of sodium pentobarbital (80 mg/kg) throughout each procedure. A 40 *μ*L suspension of pDNA (20 *μ*g) and BL (30 *μ*g) was injected into the knee joint of the ICR mice, and US exposure (frequency, 1 MHz; duty, 50%; intensity, 2 W/cm^2^; time, 60 sec) was immediately applied at the injection site. Five days after the injection, the mice were euthanized and sacrificed, and the knee joint tissue in the US-exposed area was collected and homogenized. The tissue homogenates were prepared with lysis buffer (0.1 M Tris-HCl (pH 7.8), 0.1% Triton X-100, and 2 mM EDTA). Luciferase activity was measured using a luciferase assay system (Promega, Madison, WI) and a luminometer (LB96V; Belthold Japan Co. Ltd.). Activity is indicated as relative light units (RLU) per mg of protein.

### 2.7. In Vivo Luciferase Imaging

To determine the efficiency of gene delivery, animals were divided into five experimental groups and one control group (*n* = 4 in each group). ICR mice (5 weeks old, male) were anesthetized with an *i.p.* injection of sodium pentobarbital (80 mg/kg) throughout each procedure. A 40 *μ*L suspension of pDNA (20 *μ*g) and BL (30 *μ*g) was injected into the knee joint of the ICR mice, and US exposure (frequency, 1 MHz; duty, 50%; intensity, 2 W/cm^2^; time, 60 sec) was immediately applied at the injection site. Several days after the injection, the mice were anaesthetized and *i.p.* injected with D-luciferin (150 mg/kg) (Xenogen Corporation, Calif, USA). After 10 min, luciferase expression was observed with an *in vivo* luciferase imaging system (IVIS) (Xenogen Corporation). The image of a representative of the 4 mice was used for each treatment group in this experiment.

### 2.8. Immunohistochemistry

 The gene-transfected joint tissues were preserved in 10% PFA, decalcified with EDTA, and then embedded in paraffin and sectioned. Sections (3 *μ*m thickness) were evaluated for the expression of luciferase protein by immunostaining. The sections were deparaffinized in xylene, rehydrated through graded ethanol, and equilibrated in PBS. The sections were incubated with biotin-labeled rabbit antiluciferase antibody (Cortex Biochem, San Leandro, Calif, USA). The following day, after three washes in PBS, immunoreactivity was detected using an antigoat IgG/HRP and diaminobenzidine (DAB). After color development, the joint sections were counterstained with hematoxylin and were then dehydrated, cleared, and mounted on slides.

### 2.9. In Vivo Studies

 Animal use and relevant experimental procedures were approved by Tokyo University of Pharmacy and Life Science Committee and Teikyo University on the Care and Use of Laboratory Animals. All experimental protocols for animal studies were in accordance with the Principle of Laboratory Animal Care at Teikyo University.

### 2.10. Statistical Analyses

 All data are shown as the mean ± SD (*n* = 4 or 6). Data were considered significant when *P* < .05. The *t*-test was used to calculate statistical significance.

## 3. Results and Discussion

### 3.1. Gene Transfection with Bubble Liposomes and Ultrasound Exposure into Synoviocytes In Vitro

**Figure 1:**
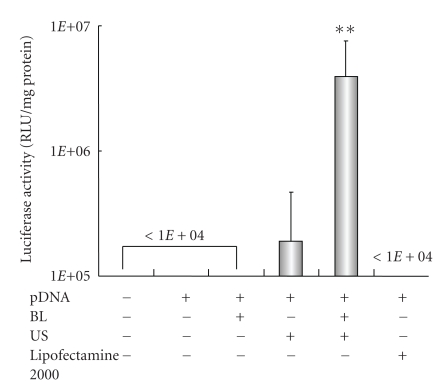
Luciferase expression in HFLS transfected with bubble liposomes and ultrasound exposure compared with Lipofectamine 2000. pDNA (pCMV-Luc) and BL were mixed together with culture medium and added to the HFLS. The cells were immediately exposed to US (frequency, 2 MHz; duty, 50%; intensity, 2.5 W/cm^2^; US exposure time, 10 sec.). The cells were washed and cultured for 2 days, and then luciferase activity was determined as described in Section 2. The transfection of pDNA by LF2000 was also performed according to the manufacturers' instructions. All data are shown as the mean ± SD (*n* = 4). **P < .05 versus other treatment groups. BL: bubble liposomes; US: ultrasound exposure.

**Figure 2:**
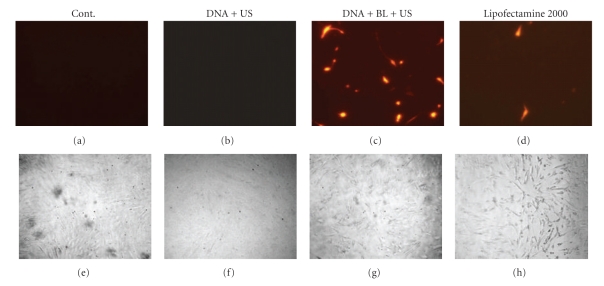
DsRed expression in HFLS transfected with bubble liposomes and ultrasound exposure compared with Lipofectamine 2000. pDNA (pDsRed-Express-N1) and BL were mixed together with culture medium and added to the HFLS. The cells were immediately exposed to US (frequency, 2 MHz; duty, 50%; intensity, 2.5 W/cm^2^; US exposure time, 10 sec.). The cells were washed and cultured for 2 days and then treated cells were examined by a fluorescence microscope original magnification X200. Transfection of pDNA by LF2000 was also performed. BL: bubble liposomes; US: ultrasound exposure. Fluorescence, (a–d); phase contrast, (e–h).

It is known that microbubbles improve cell and tissue permeability by cavitation upon US exposure [11–15]. We first tried to transfect naked pDNA (pCMV-Luc) into primary synoviocytes (HFLS), which are primary fibroblast-like cells derived from the inflamed synovial tissue of rheumatoid arthritis patients, by BL and/or US (Figure 1). As a result, luciferase activity in the group receiving a combination of BL with US exposure was 400- or 30-fold higher than that of the group treated with pDNA alone or pDNA plus US, respectively (Figure 1). For basic research, LF2000 is often used to transfect plasmid DNA or siRNA to analyze gene function in various cultured cell lines. We, therefore, compared with a commercially available transfection reagent, LF2000; however, luciferase activity was very low (Figure 1).  Figure 2 shows the transfection efficiency using DsRed expressing plasmid DNA. The numbers of DsRed-positive cells markedly increased with the combination of BL and US exposure compared to the group treated with pDNA plus US or LF2000. It may be difficult to achieve efficient gene transfection to primary cultured cells by LF2000, because a low level of transfection efficiency in human umbilical vein endothelial cells (HUVEC) was also observed (data not shown). Our previous report showed that when the intracellular localization of fluorescent-labeled siRNA in COS7 cells just after transfection with BL and US exposure is examined by confocal laser scanning microscopy (CLSM) analysis, significant cytoplasmic distribution of siRNA can be observed [25]. Consequently, we concluded that unlike the transfection method with LF2000 involving endocytosis, transfection with BL and US does not involve endocytosis, but siRNA was directly and quickly introduced into the cytoplasm by physical force. Similarly, when fluorescent-labeled plasmid DNA was delivered to COS7 cells by the combination of BL and US exposure, plasmid DNA could be also distributed in the cytoplasm and nucleus (data not shown). Therefore, these results suggested that the combination of BL and US exposure facilitated the efficient transfection of pDNA into the cells due to the induction of cavitation.

**Figure 3:**
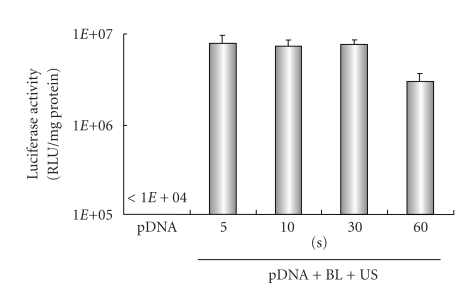
Effect of ultrasound exposure time on transfection with bubble liposomes into HFLS. pDNA (pCMV-Luc) and BL were mixed together with culture medium and added to the HFLS. The cells were immediately exposed to US (intensity, 2.5 W/cm^2^; US exposure time, 5–60 sec.). The cells were washed and cultured for 2 days, and then luciferase activity was determined. All data are shown as the mean ± SD (*n* = 4). BL: bubble liposomes; US: ultrasound exposure.

**Figure 4:**
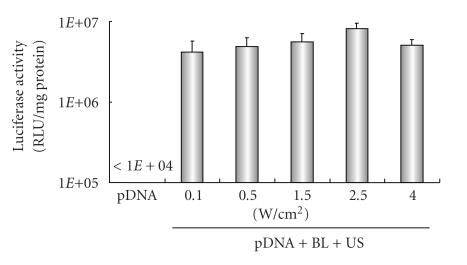
Effect of ultrasound intensity on transfection with Bubble liposomes into HFLS. pDNA (pCMV-Luc) and BL were mixed together with culture medium and added to the HFLS. The cells were immediately exposed to US (intensity, 0.1–4 W/cm^2^; US exposure time, 10 sec.). The cells were washed and cultured for 2 days and then luciferase activity was determined. All data are shown as the mean ± SD (*n* = 4). BL: bubble liposomes; US: ultrasound exposure.

Previously, our report demonstrated that gene transfection efficiency* in vitro *could be affected by increasing the US exposure time and intensity [22, 23]. We, therefore, examined the effect of US exposure time and intensity on transfection with BL into HFLS. High gene expression could be achieved by only 5 seconds of US exposure. In contrast, gene expression fell with a longer exposure time, 60 sec. (Figure 3), which might have been due to cytotoxicity. When we applied a range of US intensity (0.1–4 W/cm^2^) in transfection, US intensity of 2.5 W/cm^2^ was modestly higher than other treated groups (Figure 4). These results suggest that BL with US exposure is a useful gene delivery tool for *in vitro* transfection in synoviocytes.

### 3.2. Gene Transfection with Bubble Liposomes and Ultrasound Exposure into Joint Synovium In Vivo

**Figure 5:**
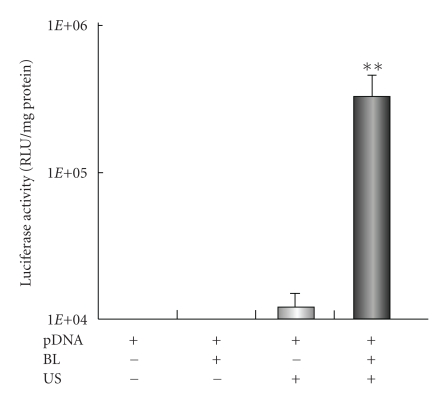
*In vivo* luciferase expression in the joint synovium after transfection with bubble liposome and ultrasound exposure. A suspension of pDNA and BL was injected into the knee joint of the mice, and US exposure (frequency, 1 MHz; duty, 50%; intensity, 2 W/cm^2^; time, 60 sec) was immediately applied at the injection site. Five days after injection, the knee joint tissue in the US-exposed area was collected and homogenized. Luciferase activity was determined. All data are shown as the mean ± SD (*n* = 4). **P < .05 versus other treatment groups. BL: bubble liposomes; US: ultrasound exposure.

**Figure 6:**
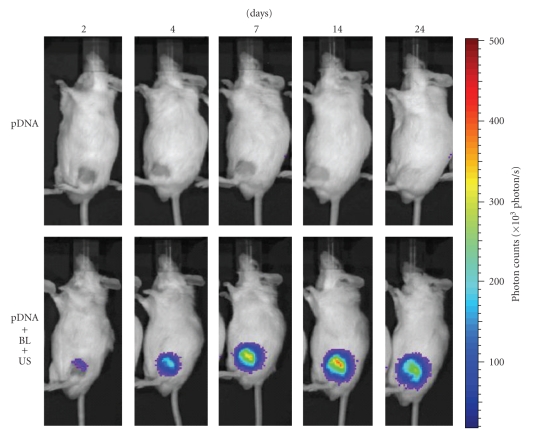
*In vivo *luciferase imaging. A suspension of pDNA and BL was injected into the knee joint of the mice, and US exposure (frequency, 1 MHz; duty, 50%; intensity, 2 W/cm^2^; time, 60 sec) was immediately applied at the injection site. Luciferase expressions after transfection into the joint treated with pDNA, or pDNA plus BL plus US exposure were observed with an *in vivo* luciferase imaging system for 2–24 days.

**Figure 7:**
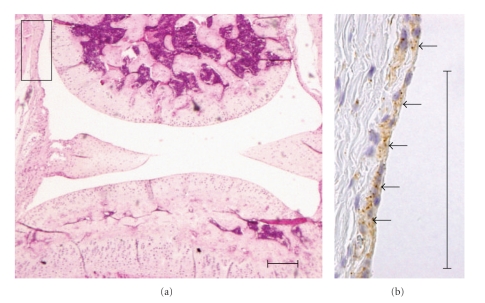
Immunostaining for luciferase in synovial fibroblast. Local gene expression in joint synovium after intra-articular gene delivery using BL and US. Seven days after treatment, the expression of luciferase protein was mostly limited to the synovial fibroblasts. (a) H&E staining in joint sections. (b) Immunohistochemical localization of luciferase (arrows). Scale bar = 100 *μ*m.

A local gene delivery system to the joint synovium by BL and US exposure may be easily applied, because the injected BL may be retained in the confined joint space and percutaneous US exposure may induce cavitations on the surface of the synovium. We, therefore, attempted to deliver pCMV-Luc, luciferase-expressing plasmid DNA, into the joint synovium of mice using BL and US and to determine the level of the gene expression. A 40 *μ*L solution of pDNA and BL was injected into the knee joint of the mice, and US exposure was immediately applied at the injection site. As a result, marked gene expression could be enhanced efficiently only with the combination of BL and US exposure when compared with other treatments (Figure 5). Exceeding our expectations, their gene expression was 500-fold higher than pDNA injection alone. We also observed luciferase gene expression area in the whole body using an *in vivo* luciferase imaging system during 2–24 days after transfection into a joint treated with pDNA, BL, and US exposure. The high level of gene expression persisted for 7–24 days after transfection using BL and US exposure (Figure 6). Gene expression was restricted to the area of US exposure. In contrast, no signal in the whole body was observed in the group with pDNA injection alone. This suggested that the combination of BL and US exposure facilitated the efficient transfection of pDNA into the joint tissue due to the induction of cavitation. We next investigated the localization of the transfected gene expression in the transfected joint by immunohistochemical analysis. This showed that the luciferase protein expression was limited to the synovial fibroblasts in the joint space. No expression was observed in other tissues such as articular cartilage (Figure 7). 

 Gene transfer into cartilage may be difficult because cavitation induction with BL and US cannot reach chondrocytes embedded in extracellular matrix in articular cartilage, leading to no transfection. However, successful gene transfection into chondrocytes might be achieved by BL and US exposure, because articular chondrocytes in RA or OA are exposed by degradation of the extracellular matrix in articular cartilage in this stage of the disease. 

It is known that cartilage degradation is the main pathological feature in OA; however, synovial factors are closely related to this progress [28], while synovitis is the main pathological feature of RA. Therefore, intra-articular gene therapy by BL and US exposure could be considered a feasible technique to deliver therapeutic proteins to suppress inflammation and destruction of the joints in RA and OA, because it could minimize extra-articular adverse effects linked to systemic injection of drugs; however, further study will be required for their assessment.

## 4. Conclusions

In this study, we showed that the combination of BL and US exposure could be an effective gene delivery method into synoviocytes *in vitro* and the joint synovium *in vivo*. In the future, this local gene delivery method with BL and US exposure might be used in non-viral gene therapy for joint disorders, such as RA and OA.
